# Is PAX-Good Behaviour Game (PAX) Associated with Better Mental Health and Educational Outcomes for First Nations Children?

**DOI:** 10.23889/ijpds.v7i3.1957

**Published:** 2022-08-25

**Authors:** Mariette Chartier, Garry Munro, Depeng Jiang, Scott McCulloch, Wendy Au, Marni Brownell, Rob Santos, Frank Turner, Leanne Boyd, Nora Murdock, James Bolton, Jitender Sareen

**Affiliations:** 1 Manitoba Centre for Health Policy, University of Manitoba; 2 Cree Nation Tribal Health Centre; 3 University of Manitoba; 4 Red River Community College; 5 Manitoba Adolescent Treatment Centre; 6 Manitoba First Nations Education Resource Centre

## Introduction

PAX, a mental health promotion approach, has been shown to decrease negative mental health outcomes and improve academic achievement. These effects have yet to be shown among Indigenous children. We evaluated PAX for improving First Nations children’s outcomes following a research process wherein community members and researchers work more collaboratively.

## Objective

Building on a long-term relationship with Swampy Cree Tribal Council, community members, First Nations leaders and researchers worked together through all phases of the project. This cluster randomized controlled trial used population-based health, social services, and education administrative data that allowed de-identified individual-level linkages across all databases through a scrambled health number. Our cohort of 725 children from 20 First Nations schools were randomized to PAX (n=469, 11 schools) or wait-list control (n=256, 9 schools). We used propensity score weighting and multi-level modeling to estimate the differences over time (2011 up to 2020) between children exposed to PAX and those who were not.

## Approach

K-anonymity processing quasi-identifiers of data may lead to ‘over generalisation’ when dealing with linkage data sets. As most linkage cases do not include all local patients and thus not all modifying data for privacy-preserving purposes needs to be used, we proposed the linkage k-anonymity (LKA) by which only obfuscated individuals in a released linkage set are required to be indistinguishable from at least k-1 other individuals in the local dataset. Considering the inference disclosure issue, we further designed the semantic-based linkage k-anonymity (SLKA) method through extending with a semantic-rule base for automatic detection of (and ruling out) risky associations from previous linked data releases. Specially, associations identified from the “previous releases” of the linkage dataset can become the input of semantic reasoning for the “next release”.

## Results

Differences in baseline characteristics were found between the two groups of children, despite the cluster randomization. After applying propensity score weights, children in the PAX group had significantly greater decreases in conduct problems (β:-1.08, standard error(se):0.2505, p<.0001), hyperactivity (β:-1.13, se:0.3617, p=.0018 ), and peer problems (β:-1.10, se:0.3043, p=.0003) and a greater increase in prosocial scores (β:2.68, se:0.4139, p<.0001) than control group children. The percentage of children in the PAX group who met academic expectations was higher than those in the control group, however, only grade 3 numeracy (odds ratio (OR):4.30, confidence interval (CI):1.34 – 13.77) and grade 8 reading and writing (OR:2.78, CI:1.01 – 7.67) met statistical significance. We found no evidence that PAX was associated with less emotional problems, diagnosed mental disorders or better student engagement.

## Conclusion

These findings suggest that PAX was effective in improving First Nations children’s mental health and academic outcomes in First Nations communities. Examining what works in Indigenous communities is crucial because approaches that are effective in some populations may not necessarily be culturally appropriate for remote Indigenous communities.

